# Children’s reporting of food insecurity in predominately food insecure households in Texas border *colonias*

**DOI:** 10.1186/1475-2891-12-15

**Published:** 2013-01-28

**Authors:** Courtney C Nalty, Joseph R Sharkey, Wesley R Dean

**Affiliations:** 1 Program for Research in Nutrition and Health Disparities, School of Rural Public Health, Texas A&M Health Science Center, MS 1266, College Station, 77843–1266, Texas, TX, USA

**Keywords:** Food security, Mexican-origin, Child self-reports, Differential reporting, Discordance, Inter-rater agreement

## Abstract

**Background:**

Food insecurity is associated with detrimental physical, psychological, behavioral, social, and educational functioning in children and adults. Greater than one-quarter of all Hispanic households in the U.S. are food insecure. Hispanic families in the U.S. comprise 30% of households with food insecurity at the child level, the most severe form of the condition.

**Methods:**

Food security discordance was evaluated among 50 Mexican-origin children ages 6–11 and their mothers living in Texas border *colonias* from March to June 2010. Mothers and children were interviewed separately using *promotora*-researcher administered Spanish versions of the Household Food Security Survey Module and the Food Security Survey Module for Youth. Cohen’s kappa statistic (κ) was used to analyze dyadic agreement of food security constructs and level of food security.

**Results:**

Eighty percent of mothers reported household food insecurity while 64% of children identified food insecurity at the child level. There was slight inter-rater agreement in food security status (κ = 0.13, *p* = 0.15). Poor agreement was observed on the child hunger construct (κ = −0.06, *p* = 0.66) with fair agreement in children not eating for a full day (κ = 0.26, *p* < 0.01) and relying on low-cost foods (κ = 0.23, *p* = 0.05).

**Conclusions:**

Mother and child-reported household and child-level food insecurity among this sample of limited-resource Mexican-origin *colonias* residents far surpass national estimates. While the level of dyadic agreement was poor, discordance may be attributable to parental buffering, social desirability in responses, and/or the age of children included in the present analysis. Future research should continue to explore how food security is understood from the perspectives and experiences of children and adolescents.

## Background

Food security requires that sustenance be available, nutritionally adequate, and acquired in socially acceptable manners [1]. Its converse, household food insecurity, is an economic and social condition characterized by reduced or unknown access to sufficient healthful and safe food or the limited ability to acquire fare in ways deemed appropriate by society. While most (85.1%) U.S. households were food secure during 2011, the remaining 14.9%, or 17.9 million, were food insecure [2].

Households with low or very low food security are considered to be food insecure. Low food security, previously known as food insecurity without hunger, occurs when individuals experience a reduction in food quality, variety, or desirability, and at times a reduction in food intake. Of the 17.9 million food insecure households in the U.S., 9.2% experienced low food security while 5.7% had very low food security in 2011 [2]. Very low food security, previously referred to as food insecurity with hunger, arises when at least one household member experiences “multiple indications of disrupted eating patterns and reduced food intake” [3]. In 2011, food insecurity at the child level was present in 10% of U.S. households with children under 18 years of age. Of households with food insecurity experienced by children, 9% had low food security among children and 1% of households had at least one child with very low food security [2].

Ordinarily, household food security status is assessed by the Household Food Security Survey Module, including three questions that pertain to the household, seven for adults, and eight questions that determine food insecurity at the child level [4-7]. One weakness of the 18-item Household Food Security Survey Module is that it identifies food insecurity at the aggregate level, and is not able to discern intra-household differences in food security among individual adults and children [8]. Furthermore, parent proxy reports of children’s food security may present an inaccurate or incomplete representation of actual experiences. Relying on guardian accounts is justified by two assumptions; first, the parent controls food resources in the home as well as how food insecurity, if present, is felt by all household members; second, individuals experience food insecurity equally as reported by the parent. However, these assumptions must be questioned, for while mothers may attempt to buffer children from the effects of household food insecurity [9,10], they may not always be fully able to protect children. It is difficult to say with certainty that all household members experience food insecurity in the same capacity, especially considering that mothers may not be fully aware of children’s experiences, resourcefulness, and actions taken to reduce the severity of food insecurity. As children who are food insecure often have poorer nutritional, educational, cognitive, developmental, and social outcomes compared to food secure children [5,11-15], measuring food insecurity in children, as reported by children, is an important next step in food security research.

There is a need to understand food insecurity from children’s perspectives and experiences [16,17]. In order to justify using child reports of food security status, researchers ascertained whether or not children could reliably report on their own experiences. Herjanic and colleagues compared mother and child reports of behavior, learning problems, psychiatric symptoms and mental status among children ages 6–16 years. With an overall 80% agreement in responses between parent and child, Herjanic et al. determined that children in this age group are capable of providing reliable information and showed that concordance is slightly improved in factual or observable information and lower in responses to questions of mental status or other internalized symptomology [18]. Herjanic and colleagues continued their analysis of parent–child agreement with a larger sample and found that mothers accurately reported behavioral symptoms while youth were better able to report their own subjective symptoms [19].

Using a modified and non-validated child food security survey, Hadley and colleagues showed that Ethiopian children ages 13–17 years were able to attest to their own food security experiences [20]. Recent research by Fram and colleagues revealed children ages 9–16 years were capable of and willing to report their own food security experiences when interviewed separately from parents [21]. While Fram’s research demonstrated that children are cognizant of food insecurity and manage their own food resources, a validated measurement tool was not used. A further limitation of Fram’s study was that researchers were unable to compare adult and child reports as a parent did not respond to questions of household food security. Connell’s qualitative research assessed children’s understanding of household food insecurity, but queried participants about other social contacts, and did not directly ask about the experiences of each child participant. Research by Connell and colleagues identified quantity, quality, psychological, and social components of children’s perceptions of household food insecurity, yet further research is needed in order to better understand food insecurity at the child level [16].

Comparing multiple accounts of food security status within a single household, even while using validated and reliable modules, might result in differential reporting between mother and child. In order to better comprehend instances of differential reporting, researchers can compare scores from two scales for concordance, known as informant agreement. Previous studies investigating the relationship between self-report and reports by others concluded that discrepancies were issues of measurement error or some flaw in study design [22]. Yet, when researchers kept methods constant for all participants and when measurement tools were shown to have good reliability, the discrepancy in reports remained high [23-25]. Therefore, discordance is not due exclusively to poor methodology, but rather that children’s experiences and perceptions may differ from those of adults.

It is important to note that the 18-item Household Food Security Survey Module ascribes a categorization of food security for all household members, and does not assign food security status to individuals within the household. In the present study, mothers report food security at the level of the household, while children, using the 9-item Food Security Survey Module for Youth, detail his or her own food security experiences and perceptions. The current research addresses a critical gap in the literature and aims to understand complexities of intra-household differences in food security. A disjuncture in mother and child food security status will prompt researchers to examine the underlying factors associated with this difference, including buffering, different food allocations for household members, cultural factors, or some other yet unrecognized association. The primary research question for the current study is as follows: How do intra-household mother and child reports of food security differ according to questions of the 18-item Household Food Security Survey Module and the 9-item Food Security Survey Module for Youth? Secondarily, using the eight child-referenced items of the Household Food Security Survey Module, how does mother-reported child food security contrast child-reports of food security when children report using the Food Security Survey Module for Youth?

## Methods

### Setting

This study was conducted in forty spatially selected *colonias* within two functionally rural areas in Hidalgo County along the Texas-Mexico border. Details of the study setting are further explained elsewhere [12]. *Colonias* are unincorporated settlements of varying sizes in which residents often build their own homes that sometimes lack sanitary living conditions, water and sewer systems, and paved roads [26]. Residents in these two areas are predominately Hispanic (93.6% and 95.6%, respectively), compared to the national rate of 15.1%, and within Hidalgo County, 82.3% of residents speak Spanish in the home [27]. These *colonias* residents encounter economic and locational disadvantages [28] and are difficult to reach by researchers and public health workers. Furthermore, research conducted in *colonias* has broad national applicability as *colonias* are prototypes for newly emerging immigrant destinations elsewhere in the U.S. [29].

### Study sample

Fifty mother-child dyads (100 study participants) were recruited by *promotora*-researchers (certified community health workers trained in research methods who are residents of the community and native Spanish speakers) for a cohort study. Twenty-five dyads from western Hidalgo County *colonias* and twenty-five dyads from eastern *colonias* were enrolled in the study. *Promotora*-researchers delivered letters of invitation to participants’ homes. Inclusion criteria necessitated that a child between the ages of 6–11 years reside in the household. Interviews with children led by *promotora*-researchers indicated that *colonia*-dwelling Mexican-origin children in this age group understood concepts of food security and were willing to report their experiences. Mothers were advised on the nature of the study (assessments, confidentiality, and financial incentive) and provided consent for herself and her child while children provided assent to participate in the study. All materials and protocols were approved by the Texas A&M University Institutional Review Board.

### Data collection

Data were collected in Spanish between March and June 2010 in participants’ homes by *promotora*-researchers. Mothers provided demographic covariates (age, education, marital status, self-identified race/ethnicity, country of birth, household size, total income, work status, and enrollment in nutrition assistance programs). Household food security was assessed through mother reports of the United States Department of Agriculture (USDA) Economic Research Service 18-item U.S. Household Food Security Survey Module, validated for use with Hispanic populations [30]. Children completed the 9-item Child Food Security Survey Module [31]. Overall household and child-level food security were assessed using the complete 18- and 9-item surveys, respectively. However, mother-child agreement for individual food security constructs was limited to the nine items that had a corresponding item on the child food security module; these constructs included 1) worrying about food supplies, 2) running out of food supplies, 3) using low cost foods for children’s meals, 4) lack of a balanced diet for children, 5) children not eating enough, 6) reducing children’s portion size, 7) children skipping meals, 8) children experiencing hunger, and 9) children going a full day without eating. Mothers’ reports of household food security, along with child food security derived from the eight child-referenced items of the Household Food Security Survey Module, were each compared with child reports using the child module. See Table 1 for full detail of comparative question sets. For mothers and children, a three month reference period was used to assess food security. *Promotora*-researchers interviewed children separately from mothers to avoid parental influence and bias. Additionally, children self-reported age at time of survey. Both versions of the USDA Economic Research Service survey modules were translated from English to Spanish and then back-translated by a certified multi-lingual translator and reviewed by a team of native Spanish speakers. With all study participants, *promotora*-researchers assisted with survey administration by reading aloud questions and recording responses on paper copies.

**Table 1:** Food security constructs used to compare mothers’ and children’s responses

**Food security construct**	**Household Food Security Survey Module Question**	**Food Security Survey Module for Youth Question**
**Worry about food supplies**	HH2) “(I/We) worried whether (my/our) food would runout before (I/we) got money to buy more.” Was that often true, sometimes true, or never true for (you/yourhousehold) in the last 3 months?	1) Did you worry that food at home would run out before your family got money to buy more?
**Run out of food supplies**	HH3) “The food that (I/we) bought just didn't last, and(l/we) didn't have money to get more.” Was that often,sometimes, or never true for (you/your household) inthe last 3 months?	2) Did the food that your family bought run out, and you didn’t have money to get more?
**Low cost foods for children**	CH1) “(I/we) relied on only a few kinds of low- cost food to feed ((my/our) child/the children) because (I was/we were) running out of money to buy food.” Was that often, sometimes, or never true for (you/your household) in the last 3 months?	3) Did your meals only include a few kinds of cheap foods because your family was running out of money to buy food?
**Lack of a balanced diet for children**	CH2) “(I/ We) couldn’t feed ((my/our) child/the children) a balanced meal because (I/we) couldn’t afford that.“ Was that often, sometimes, or never true for (you/your household) in the last 3 months?	4) How often were you not able to eat a balanced meal because your family didn’t have enough money?
**Children not eating enough**	CH3) “My/Our child was/The children were) not eatingenough because (I/we) just couldn't afford enoughfood.” Was that often, sometimes, or never true for(you/your household) in the last 3 months?	5) Did you have to eat less because your family didn’t have enough money to buy food?
**Reduce children’s portion sizes**	CH4) In the last 3 months, did you ever cut the size of(your child’s/any of the children’s) meals because therewasn't enough money for food?	6) Has the size of was your meals been cut because your family didn’t have enough money for food?
**Children skip meals**	CH5) In the last 3 months, did (CHILD’S NAME’ any of the children) ever skip meals because there wasn’t enough money for food?	7) Did you have to skip a meal because your family didn’t have enough money for food?
**Children are hungry**	CH6) In the last 3 months, (was your child/were the children) ever hungry but you just couldn’t afford more food?	8) Were you hungry but didn’t eat because your family didn’t have enough food?
**Children go full day without eating**	CH7) In the last 3 months, did (your child/any of the children) ever not eat for a whole day because there wasn’t enough money for food?	9) Did you not eat for a whole day because your family didn’t have enough money for food?

### Analysis

Data were entered from hard copy into Access databases and verified for accuracy by an independent coder. Affirmative responses on the household scale (“Often true” or “Sometimes true”) were coded as Yes while negative responses (“Never true”) were coded as No. Similarly, response options on the children’s scale were based on frequency (“A lot,” “Sometimes,” or “Never,”); for analysis the two affirmative responses were coded as Yes while “Never” was coded as No. Scoring for the food security assessments was in accordance with USDA Economic Research Service recommendations. Affirmative responses were summed as ordinal variables; the 18-item survey was scored in the following manner: 0 = high food security, 1–2 = marginal food security, 3–7 = low food security, 8–18 = very low food security. Child food security as determined from the household module was computed through the summation of affirmative responses on items CH1-CH7 and scored in the following manner: 0 = high food security among children, 1 = marginal food security among children, 2–4 = low food security among children, 5–8 = very low food security among children [32]. The child-reported 9-item survey was scored in the following manner: 0 = high food security, 1 = marginal food security, 2–5 = low food security, 6–9 = very low food security [17,31]. Complete food security data were available on 100% of study participants.

Descriptive statistics (median, standard deviation, number and percent) for mother-reported variables (age, country of birth, education, race/ethnicity, marital status, household size, household monthly income, and utilization of nutrition assistance programs, including Supplemental Nutrition Assistance Program (SNAP), Supplemental Nutrition Program for Women, Infants, and Children (WIC), and school nutrition programs (School Breakfast Program [SBP] and National School Lunch Program [NSLP])) as well as the child-reported variable (age) were calculated.

The amount of agreement, or precision, between mother and child reports of food security status and individual food security constructs was assessed using Cohen’s kappa (κ) statistic, which provides the amount of agreement between two unique raters after considering chance agreement [33]. Terminology for describing the strength of agreement originated from work by Landis and Koch, who ascribe κ of <0.00 as “poor,” 0.00–0.20 as “slight,” 0.21–0.40 as “fair,” 0.41–0.60 as “moderate,” 0.61–0.80 as “substantial,” and 0.81–1.00 as “almost perfect” [34]. While other measures of agreement, including intraclass correlation coefficient, exist, kappa was chosen for the current analysis as it is a preferred method for nominal (food security outcome variable was coded as 0 = food secure, 1 = food insecure), rather than ordinal, outcomes [33]. To further investigate the role of intra-household child demographics in food security agreement, the number of children in each age group (according to cut-points recommended by the Centers for Disease Control and Prevention) as well as the total number of children in each home, were cross tabulated by agreement; Fisher’s Exact Tests assessed significant associations among the limited sample size. Information on power analysis for discordance measures using kappa statistics is limited, although it is estimated that a sample size of 30 with two unique raters is sufficient given an expected kappa of 0.40 or greater [35]; in the present analysis, the sample size of 50 dyads may accommodate lower rates of agreement. All analyses were performed using Stata Statistical Software: Release 11 (College Station, TX: Stata Corp, 2009) and *p*-values less than 0.05 were deemed statistically significant.

## Results

Table 2 displays demographic characteristics of the sample. Median age (SD) of mothers was 34.5 years (± 6.9) while child median age was 8.5 years (± 1.3). Ninety-two percent of mothers were born in Mexico, with the remainder born in the United States, and mothers completed a median nine years (± 2.5) of school. Median household size was six adults and children (± 1.5, range: 3–10) and median number of children living in the home was 3.5 (± 1.2, range: 1–6). In this sample, 96% of children were enrolled in SBP, 88% of families utilized SNAP, 58% of families relied on WIC, 58% of children were enrolled in NSLP, and 32% of families were enrolled in all four nutrition assistance programs.

**Table 2:** Select demographic characteristics of mother-child dyads (n = 100)

	*Median ± SD*	*Range*	*N (%)*
Mother age, y	34.5 ± 6.9	20 - 55	
Child age, y	8.5 ± 1.3	6 - 11	
Mother education, y	9.0 ± 2.5	1 - 16	
Household size	6.0 ± 1.5	3 - 10	
Children in household	3.5 ± 1.2	1 - 6	
Child sex			
Female			31 (62)
Mother country of birth			
Mexico			46 (92)
USA			4 (8)
Mother race/ethnicity			
Hispanic			16 (32)
Mexican			34 (68)
Nutrition assistance			
SNAP			44 (88)
WIC			29 (58)
School breakfast			48 (96)
School lunch			29 (58)

Equivalent mother and child-reported food security constructs from the two modules were used to analyze differential reporting. Analyses revealed a striking difference in reporting, with poor agreement on one, slight agreement on six, and fair agreement on two constructs. The poorest agreement was on the construct of child hunger (κ = −0.06, *p* = 0.66). Fair agreement was observed among the constructs of children going a full day without eating (κ = 0.26, *p* = 0.003) and using low cost foods for household meals (κ = 0.23, *p* = 0.05). Furthermore, 34% of children reduced portion sizes and 30% of children skipped meals, compared to affirmative responses by 12% of mothers reporting on behalf of their children for each construct (see Table 3).

**Table 3:** Proportion of mothers and children responding affirmatively to food security constructs

**Food security construct**	**Affirmative response**		**Kappa ( *p *-value)**	**Strength of Agreement**
	**Mother n (%)**^**1**^	**Child n (%)**^**2**^		
Worry about food supplies	46 (92)	25 (50)	0.08 (0.15)	slight
Run out of food supplies	30 (60)	23 (46)	0.02 (0.45)	slight
Low cost foods for children	30 (60)	27 (54)	0.23 (0.05)	fair
Lack of a balanced diet for children	29 (58)	20 (40)	0.19 (0.08)	slight
Children not eating enough	17 (34)	16 (32)	0.05 (0.36)	slight
Reduce children’s portion sizes	6 (12)	17 (34)	0.10 (0.19)	slight
Children skip meals	6 (12)	15 (30)	0.14 (0.13)	slight
Children are hungry	2 (4)	4 (8)	−0.06 (0.66)	poor
Children go full day without eating	1 (2)	6 (12)	0.26 (0.003)	fair

^1^ Affirmative response is a combination of “often true” and “sometimes true”.

^2^ Affirmative response is a combination of “a lot” and “sometimes”.

Eighty percent of mothers reported household food insecurity while 64% of children experienced food insecurity at the child level. Mothers were more likely to report low or very low food security at the household level (58 and 22 percent, respectively) than were children to report low or very low food security at the child level (44 and 20 percent, respectively). Furthermore, the observed agreement between mother and child self-reports of the two-level food security categorization was 64%, or “slight” (κ = 0.13, *p* = 0.15). The greatest mother-child agreement was noted among food insecure families; thirty-two children reported food insecurity at the child level, while 27 mothers reported household-level food insecurity. Of the remaining 18 children, who by self-reports were food secure, only five mothers agreed and reported household food security (See Table 4). Among discordant pairs, small sample *χ*^2^ analyses revealed no significant differences in food security when considering the total number of children or the ages of all children in the household. Comparing mothers’ reports of child food security with child reports of child food security revealed 56%, or slight agreement, between the binary outcomes (κ = 0.09, *p* = 0.26).

**Table 4:** Agreement in mother and child self-reports of food security status

**Participant**					
	**Child**				
	**Level of food security**	**Food secure**^**1**^	**Food insecure**^**2**^	**Total**	**%**
**Mother**	**Food secure**^**1**^	5	5	10	20
	**Food insecure**^**2**^	13	27	40	80
	**Total**	18	32	50	
	**%**	36	64		
**Kappa ( *p *-value)**		0.13 (0.15)			
**Strength of Agreement**		Slight			

^1^ High food security and marginal food security.

^2^ Low food security and very low food security.

## Discussion

The aim of the present study was to analyze inter-rater agreement of food security among a sample of Mexican-origin children age 6–11 years and their mothers living in Texas border *colonias*. In this research, the first of the authors’ knowledge to contrast mother and child accounts of food security, analyses revealed differential response rates in food security status among dyads. The prevalence of mother-reported household food insecurity (80%) surpassed children’s reports of food insecurity (64%), indicating only slight inter-rater agreement. The prevalence of mother-reported child food insecurity (56%), as determined through the household scale, was less than the prevalence of child food insecurity as reported by the children themselves (64%). Therefore, if we consider children’s reports of their own food insecurity to be reliable, then there is some evidence that mothers may not be fully protecting children from the effects of household food insecurity. Thinking of food security as distinct from food insecurity, children in this sample of Mexican-origin *colonias* residents were reliable reporters of their own food insecurity as it correlates with household and mother-reported child-level food insecurity, but children’s reports of child-level food security did not correspond well with mothers’ reports of household or child level food security. Thus, there is some evidence that children as young as six years of age may possess some ability to reliably report instances of food insecurity, although further cognitive testing among a larger sample is encouraged.

In the current study, mothers reported food security for the household, including other adults and multiple children in the home, while children reported their unique experiences of food security. Discordance may therefore arise from mothers’ responses for all children in the home (as the module instructs) and not solely that of the index child who completed the youth module. This becomes of particular interest when considering demographics of children in the home, as teenagers may buffer younger children from the effects of food insecurity. However, among the discordant pairs, there were no statistically significant differences by family composition, neither by the overall number of children living in the household nor by the number of children within each five-year incremental age group. Consequently, for this limited sample of Mexican-origin *colonias* residents, differential food security reporting was not influenced by household structure. Still, alternate reasons for discordance may be at play, including social desirability inherent in mothers’ reports or parental buffering of children, which may diminish reports of household and child-level food insecurity, respectively.

Previous food security research using the U.S. Household Food Security Survey Module has relied almost exclusively on mothers’ perceptions of children’s food security and is unable to provide individual-level information [4-7]. However, research utilizing child reports of food security is emerging. Children’s very low food security, as reported by Mexican-origin children ages 6–11 years, was associated with significantly higher intake of total energy, fat, and added sugar as compared to food secure children in a recent finding by Sharkey and colleagues [12]. Additionally, recent qualitative research has begun the process of understanding the unique ways in which children understand, cope with, and attempt to alleviate the symptoms of food insecurity [16,21,36].

Prior research established that children age 6–16 years can be reliable reporters [18,19] and at ages as young as nine can share valuable information regarding their food security [16,21]. Herjanic and colleagues detected concordance between mother-child reports of observable behavioral symptomology among children and less agreement among children’s internalized emotions [19]. The results of the present study indicate overall poor agreement of food security status among mother and child reports but particularly poor accord among subjective experiences. In fact, mothers and children had the strongest agreement in the construct of children not eating for a full day, which may be more likely to be noticed than internal emotions, such as worrying about food supplies. The constructs in which children more frequently reported an occurrence than mothers were reducing portion sizes, skipping meals, or being hungry, which all showed poor agreement. While mothers’ responses may be influenced in part by social desirability, it could also be that these events were not as frequently observed by mothers and thus may demonstrate actions taken by children to preserve food for a lean period. Numerous recently published articles have compared parent-proxy and child reports of children’s experiences and perceptions of medical conditions or overall quality of life. One review article of nineteen quality of life subscales (including data on children ranging in age from 5–20 years) used intraclass correlation coefficients to determine interrater agreement and concluded that parent–child agreement ranged from poor to good [37]. In research not included in the review article, Creemens and colleagues found low intraclass correlation between parents and children ages 5.5–8.5 years on quality of life reporting, and suspect that level of disagreement may be a result of child age, analysis methodology, the domain analyzed, or parent’s own quality of life [38]. Finally, qualitative techniques were used among 15 parent–child pairs to discern the underlying reasons for discordance in reporting of quality of life scales in a study by Davis and colleagues. Research revealed that among a sample of 5–11 year old children and their parents, discordance may be a result of different reasoning methods and response styles [39]. Overall low correlation among parent–child agreement in child health-related quality of life is consistent with the findings of the present study of agreement in food security status. Research in other fields offers some basis of comparison, yet there is a dearth of literature on agreement in parent–child food security reporting.

There are several strengths to this original research. This is the first study of which the authors are aware that utilized children’s self-reports of food security constructs and level of food security contrasted with reports provided by mothers. This study answers the call to consider the perspective of the child in analyzing the prevalence and correlates of food insecurity [16,17,21]. Second, this research provided valuable information about a sample of hard-to-reach, limited-resource Mexican-origin families living in Texas border *colonias*. As the Hispanic population in the U.S. is growing, and is expected to comprise 29% of the population by 2050 [38,40], and South Texas *colonias* may represent an archetype for new-immigrant destinations elsewhere in the U.S. [29], understanding food insecurity among this population is imperative to decreasing the burden of health disparities, especially those related to nutrition. Third, data were collected using Spanish-language surveys and interviews within participants’ homes and obtained by trained *promotora*-researchers who reside within the study locale. Relying on local *promotora*-researchers as data collectors and community advocates established trust among this sample. Additionally, the food security instruments used in the present research were developed with the USDA Economic Research Service, have high internal validity, and are approved for use both among children and Hispanic populations [16,17,30]. Finally, complete food security data were collected on 100% of participants.

Yet, there are limitations that warrant mention. First, the study included 50 dyads and utilized a cross-sectional research design. Thus, there is limited generalizability to other populations. A larger study in a broader locale may improve researchers’ understanding of differential reporting among mothers and their children. Second, the age of children in this study may have affected reporting. While the Food Security Survey Module for Youth was designed for use with children age 12 years and older, the children in the present study were 6–11 years of age. Interviews with *promotora*-researchers indicated that children in this age group understood food security and showed willingness to report while other research demonstrated the ability of children to self-report at as young as six years of age [18-20]. Although the present study’s child sample is within the acceptable age range of children’s reliability to self-report conditions, future researchers may elect to focus on an adolescent population. Because the household food security module asks mothers to report the experiences of all children in the home, while one child reported his/her individual experiences, researchers were unable to directly compare individual-level discordance. This additionally hindered the researchers’ understanding of food security among several children within a household. Finally, the reference period for the food security modules was the previous three months and not 30 days, which prior research has indicated may be more reliable when surveying children [31].

## Conclusions

The Mexican-origin population in the United States is growing rapidly, with a 54.1% increase from 2000 to 2010. Furthermore, the nearby four-city area of McAllen-Edinburg-Pharr-Mission, Texas is comprised of 96.8% residents of Mexican origin [41]. Due to the detrimental nutritional, behavioral, social, academic, and health-related outcomes among children living in food insecure households [5,11-15], and because school-based nutrition programs may not be adequate in addressing children’s nutritional needs [12], children’s food insecurity among the increasing Mexican-origin population in the U.S. cannot be overlooked. This research addresses a gap in children’s food security literature and sheds light onto a difficult-to-reach population of Mexican-origin families living in Texas border *colonias*. Future research endeavors ought to assess food security from children’s perspectives, among a larger and slightly older sample, in accordance with the recommendations by the USDA Economic Research Service.

## Competing interests

The authors declare that they have no competing interests.

## Authors’ contributions

JRS designed the research study, and worked on the development of the instruments and the protocol for collection of data. CCN developed the research question and conducted the analysis. CCN, JRS, and WRD wrote the first draft of the paper. CCN, JRS, and WRD read and approved the final manuscript.
